# Synthesis, characterization and biological profile of some new dihydropyrimidinone derivaties

**DOI:** 10.1016/j.heliyon.2024.e41152

**Published:** 2024-12-12

**Authors:** Madiha Kanwal, Humaira Nadeem, Sumra Malik, Saima Maqsood

**Affiliations:** Department of Pharmaceutical Chemistry, Riphah Institute of Pharmaceutical Sciences, Riphah International University, Islamabad, Pakistan, 44000

**Keywords:** Anti-Bacterial, Anti-Fungal, Anti-oxidant, Anti-inflammatory, Cytotoxicity, Dihydropyrimidinones. derivatives

## Abstract

**Objective:**

The rise of drug-resistant bacteria, viruses, and fungi has prompted the search for new drugs without cross-resistance to current treatments. As a result, the aim of this research was to synthesize various types of dihydropyrimidinones heterocyclic compounds and screened them for their antibiotic properties.

**Methodology:**

Newly synthesized dihydropyrimidinone derivatives were characterized spectroscopically using proton NMR (^1^HNMR), and FT-IR. These substances were then subjected to molecular docking studies via Auto dock Vina software to determine their affinity for binding to proteins from different bacterial strains including (*Staphylococcus epidermidis* (S. epidermidis), *Staphylococcus aureus* (*S. aureus*), *Mycobacterium luteus* (M. luteus), *Salmonella typhi* (S.typhi), *Bacillus subtilis* (B. subtilis), *and Escherichia coli* (*E. coli*) and fungal (*Candida glabrata* (C. glabrata), *Candida albicans* (C. albicans), and *Saccharomyces cerevisiae* (*S. cerevisiae*) strains. Also in-vitro anti-fungal, anti-bacterial and anti-oxidant activity was performed by using ager well diffusion method and DPPH assay respectively. Moreover, the *In-vivo* biological evaluation of these derivatives was conducted by using carrageenan-induced hind paw model. The cytotoxicity profile of the synthesized derivatives was done via *in-vitro* MTT assay.

**Results:**

All newly synthesized derivatives were confirmed via the multiple spectroscopic analysis techniques. All derivatives showed good binding affinities against the multiple targeted protein with. Compound 4c exhibited hightest potential with −10 kcal/mol against bacterial strains. 4b showed best antifungal potential with −10.8 kcal/mol binding affinities. For *Bacillus subtilis* compound 4b and 4c performed best with 17 mm ± 2.21. for anti-fungal activity against *Candida glabrata,* amongst the five newly formed compounds, 4a showed best activity with 19 mm ± 1.22 The analogue 4b exhibited best anti-oxidant potential with 63.85 ± 1.39. Compound 4a and 4b showed highest anti-inflammatory potential with 1.011 mm ± 0.247 mg/kg and 1.447 ± 0.212 mg/kg in countering inflammation by targeting toll-like receptor activation and reduce the inflammation in hind paw edema. The selected derivatives exhibited no toxicity profile with 99 % and 98 % cell survival rate using compound 4a and 4b.

**Conclusion:**

Research has been done on the multiple biological activities of dihydropyrimidinones derivatives but the innovation on MDR is still pending. New dihydropyrimidinone derivatives were developed as agents to combat drug resistance. The results of these studies showed that newly synthesized dihydropyrimidinone derivatives are remarkably effective not only as anti-biotic agents but also counter inflammation caused by carrageenan resulting from the activation of the Toll-like receptors (TLRs) signaling pathway along with the non-toxic effect. So it is concluded that the recently synthesized new dihyropyrimidinone derivatives are highly effective antimicrobial derivatives with non-toxic effects on human cell lines.

## 1 Introduction

The resistance to bacterial strains is the most emerging problem throughout the world. Normally the bacteria have the capacity to modify themselves according to the environment and create the resistance against the anti-bacterial drugs. Also the multidrug-resistant bacteria (MDR) cause the intense infection around the world which created the need to develop new antibiotics [1]. This method may be able to overcome the limitations of traditional drug development and leads to the breakthrough for the innovative planning in drug development. This may also provide potential for the treatment of a number of diseases. The change of this strategy may lead to the opening of new doors for the medicinal chemist in the field of drug development [2]. Pyrimidine is an important part of DNA and RNA and plays a vital role in many biological processes. It has been part of multiple natural compounds such as vitamins, liposaccharides, and many commercially available antibiotics. In Organic synthesis, pyrimidine nucleus also contributed a lot [3]. It is also a considerable moiety as a function of antibacterial, antibiotic, agrochemical, veterinary products, and as a cardiovascular agent. Several research studies revealed that its biological background is very strong enough as its derivatives showed different activities like, anti-inflammatory [4], anti-viral [5], anti-avian [6], analgesic [7], influenza virus (HSN1) [5], hepatitis A virus (HAV) [8], anti-arrhythmic agent [9], serotonin 5-HT_6_ receptors [10]. Derivatives containing pyrimidine moiety also involve in platelet aggregation inhibitor property and in anti-Parkinson s inhibitor activity [12]. Multicomponent reactions (MCR) are one-pot process in which one or more than one reactant are combined by a single reaction in a vessel to form a product. These MCR are most important in the field of medicinal chemistry due to their contributions likewise, molecular complexity, atom economy, convergence, and selectivity. Dihydropyrimidinones derivatives are one of the examples of synthesized compounds by this reaction method. MCR are a special type of useful organic reactions which consist of three or more reactants to form a required product. Combine synthesis privileges upon single step reactions in respect to the time, speed, yield and reproducibility of product. Due to this reason MCR created a superior tool for diversity oriented and complexity –generating synthesis of heterocyclic compounds [11]. According to the literature review, rare-earth coordination polymers (CPs) have been reported as good antimicrobial agents including the facile synthesis of ternary lanthanum CPs at room temperature which were later on fond to be potent anti-microbial and antitumor agents [12]. Dihydropyrimidinones are well known Beginelli reaction components and important for their diverse biological activities including anti-tumor, antihypertensive, calcium channel blockers, α-1a-adrenergic blockers. On the other side alkaloid containing dihydropyrimidinones nucleus also possess different biological spectrum [13]. Between 1990 and December 31st, 2016, 115 articles represented biological activities and toxicity of DHPM derivatives. Few of these articles are involved in *in-vivo* studies. Antitumoral (43 articles), anti-inflammatory (12 articles), anti-bacterial (20 articles) represents the data for the said compounds [14]. Oxidative stress is associated with the imbalance of multiple pathways like the production of free radicles and the extent of body antioxidants. Several body disorders like inflammation and pain is associated with this imbalance [15,16]. Inflammation is the core process required as defense mechanism in animals and humans against several external injuries [17,18]. Inflammation may be categories as acute [19] and chronic [20]. Multiple neurodegenerative disorders, cancer, cardiovascular disorders are associated with chronic inflammation [21]. In inflammation white blood cells, leukocytes, basophils, neutrophils, eosinophils, plasma and fluids at the site of inflammation change their location [22]. Different mediators like histamine, prostaglandins, leukotrienes, oxygen and nitrogen related free radicles are released by immune cells involved in the process of inflammation [23]. Cyclooxygenase-1 & cyclooxygenase-2 (COX-1 & COX-2) are the key enzymes play an important role in the process of inflammation [[24], [25], [26], [27], [28]]. Pyrimidine nucleus containing derivatives represents anti-oxidant [29] anti-inflammatory [4,7,30], anti-bacterial [[31], [32], [33], [34]], anti-fungal [35,36] potential. Due to advances in multifunctional technology have led to increase demand of multifunctional products. From the literature review it is evident that a lot of research wok has been done so far on the dihydropyrimidinones derivatives but still few areas are uncovered. The basic anti-bacterial, anti-fungal and cytotoxicity studies need to be done to ensure the *in-vitro* biological profile of these derivatives. The aim of the current manuscript is to design, synthesize, and screen the *in-vitro* biological activities including anti-bacterial, anti-fungal, anti-oxidant activity, and finally to evaluate anti-inflammatory potential *(in-vivo*) by using albino mice. Also the cytotoxicity studies was done via MTT assay to evaluate the toxic effect of the synthesized derivatives (see Fig. 4).

## 2 Result and discussion

### 2.1 Chemistry

#### 2.1.1 Compound's design

**Scheme 1:**
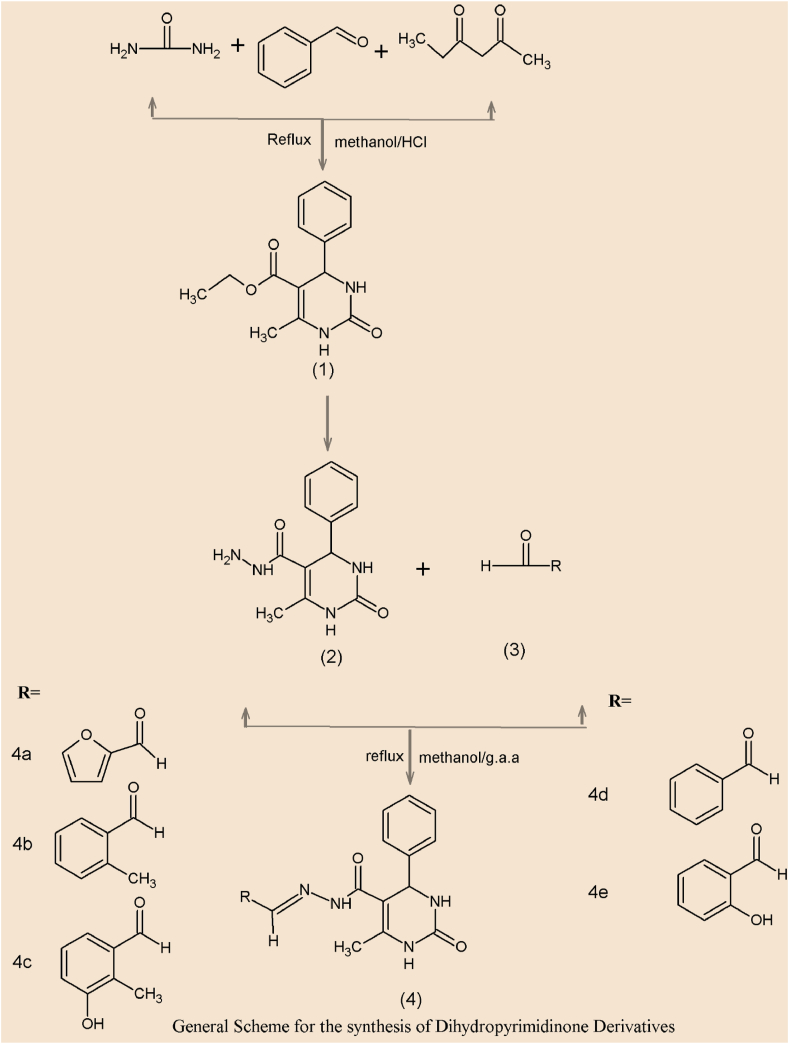
General scheme for the synthesis of dihydropyrimidinone derivatives.

New dihydropyrimidinone derivatives were synthesized using traditional Beginelli reaction by reflexing mixture of benzaldehyde, urea, and ethyl acetoacetate using methanol as solvent and concentrated HCl as catalyst. This led to the synthesis of **Ethyl 6-methyl-2-oxo-4-phenyl-1,2,3,4-tetrahydropyrimidine-5-carboxylate (1).** The resulting compound obtained was later dissolved in ethyl alcohol for the synthesis of hydrazide. Hydrazine hydrate was added to the solution and the reaction mixture was refluxed again. The white-colored product **(2)** obtained was filtered and recrystallized using ethanol [37]. The carbohydrazide was then dissolved in ethanol to get a clear solution. Different substituted aryl aldehydes dissolved in ethanol were added to this solution to synthesize different dihydropyrimidinone products (Scheme 1) [38]. To ensure the purity of the synthesized compounds and to track the development of the reaction, TLC was utilized at every stage. All of the derivatives' structures were determined using IR and 1H NMR spectroscopic data, and it was discovered that they were all consistent with the molecular structures that had been proposed.

Final dihydropyrimidinone derivatives (4a-4e) synthesized by coupling of aryl aldehyde with hydrazide.

### 2.2 Molecular docking studies

A docking study of newly synthesized compounds 4a – 4e against target proteins involved in antibacterial and anti-fungal mechanisms was performed to extend the knowledge via Autodock vine 4.2.6. The X-ray crystal structure of the bacterial proteins used were the following: Transcription regulator from Staphylococcus epidermidis (PDB ID: 3kp5), oxidoreductase enzyme from *Staphylococcus aureus* (PDB ID: 5yh5), hydrolase enzyme from Salmonella Typhi (PDB ID: 2wnw), MetH C-terminal fragment form *Escherichia coli* (PDB ID: 1k7y), sirohydrochlorin ferrochelatase (SirB)- a biosynthetic protein-from Bacillus subtilis (PDB ID: 5zt7), and transferase enzyme form Mycobacterium luteus (PDB ID: 4ewp) (Table 2). Fungal proteins used for docking were the following: dihydrofolate reductase enzyme from Candida glabrata (PDB ID: 3eej), membrane protein from Candida albican (PDB ID: 7rjb), transferase enzyme particularly SNF-1 protein kinase complex from *Saccharomyces cerevisiae* (PDB ID: 3te5) were retrieved from the online protein data bank (Table 3).

**Table 1:** Physical characteristics of synthesized compounds.

Compounds	Mol. Formula	Melting Point (^0^C)	Physical State	Percentage Yield	R_f_ Value
IV a	C_18_H_16_N_4_O_4_	200–210 °C	Solid	40 %	0.55
IV b	C_21_H_20_N_4_O_3_	194 °C	Solid	45 %	0.59
IV c	C_21_H_20_N_4_O_4_	195 °C	Solid	55 %	0.60
IV d	C_20_H_18_N_4_O_3_	190–192 °C	Solid	40 %	0.56
IV e	C_20_H_18_N_4_O_4_	188-189 °C	Solid	30 %	0.61

**Table 2:** Table showcases amino acids involved in making hydrogen bonds and respective binding energies of dihydropyrimidinone derivatives (4a - 4e) with targeted proteins of selected bacterial strains.

Bacterial Proteins					
Protein	PDB ID	Compound	Hydrogen Bond	Amino acid	Binding Energy (kcal/mol)
Transcription regulator from Staphylococcus epidermidis	3kp5	**4a**	2	Asn 20, Arg 110	−8.9
		**4b**	1	Arg 110	−9.4
		**4c**	2	Gln 61, Arg 110	−10
		**4d**	1	Arg 110	−9.7
		**4e**	2	Asn 20, Arg 110	−9.5
		**Tetracycline**	6	Asn 20, Thr 21, Glu 39, His 42, Lys 60, Gln 61	−9.2
Oxidoreductase enzyme from *Staphylococcus aureus*	5yh5	**4a**	4	Arg 248, Gln 404, Ser 478, Thr 480	−8.4
		**4b**	1	Arg 43	−9.1
		**4c**	2	Gln 404, Gln 496	−9.4
		**4d**	3	Arg 140, Asn 253, Thr 405	−8.8
		**4e**	1	Arg 43	−9.4
		**Tetracycline**	3	Gln 404, Trp 406, Tyr 410	−8.2
Hydrolase enzyme from Salmonella Typhi	2wnw	**4a**	1	Ser 388	−9.5
		**4b**	3	Ser 276, Asn 391, Glu 395	−9.8
		**4c**	3	Ser 276, Lys 322, Ser 388	−10.2
		**4d**	2	Gln 279, Pro 286	−8.5
		**4e**	2	Ser 276, Gln 279	−10
		**Tetracycline**	4	Asp 273, His, 311, Asp 390, Arg 412	−8.6
MetH C-terminal fragment form *Escherichia coli*	1k7y	**4a**	1	Lys 1148	−8.2
		**4b**	1	Ala 860	−9.1
		**4c**	0	–	−8.4
		**4d**	1	Arg 1134	−8.8
		**4e**	3	Asp 760, Ile 761, Ser 1176	−8.6
		**Tetracycline**	2	Arg 1094, Ser 1176	−8.3
Sirohydrochlorin ferrochelatase (SirB)- a biosynthetic protein- form Bacillus subtilis	5zt7	**4a**	2	Met 1, Arg 116	−8
		**4b**	1	Arg 134	−8.6
		**4c**	0	–	−8.8
		**4d**	0	–	−8.5
		**4e**	4	Met 1, Arg 116, Ile 224, Phe 226	−8.5
		**Tetracycline**	5	Gln 3, Glu 26, Arg 96, Ala 243, Asn 244	−8.6
Transferase enzyme form Mycobacterium luteus	4ewp	**4a**	1	Arg 47	−7.5
		**4b**	2	Gly 240. Val 297	−8.3
		**4c**	4	Arg 47, Gly 240, Val 297, Asn 323	−8.3
		**4d**	1	Arg 299	−7.1
		**4e**	2	Glu 257, Tyr 342	−8.9
		**Tetracycline**	4	Ala 21, Arg 23, Glu 72, Arg 280	−8.2

**Table 3:** Table showcases amino acids involved in making hydrogen bonds and respective binding energies of dihydropyrimidinone derivatives (4a - 4e) with targeted proteins of selected fungal strains.

Fungal Proteins					
Protein	PDB ID	Compound	Hydrogen Bond	Amino acid	Binding Energy (kcal/mol)
Dihydrofolate reductase enzyme form Candida glabrata	3eej	**4a**	2	Ala 11, Thr 58	−9.3
		**4b**	1	Ala 11	−8.4
		**4c**	3	Ala 11, Thr 58, Ser 61	−10.1
		**4d**	2	Thr 58, Ser 61	−10
		**4e**	2	Thr 58, Ser 61	−10.3
		**Nystatin**	5	Arg 99, Thr 103, Arg 128, Asp 135, Arg 165	−9.9
Membrane protein from Candida albican	7rjb	**4a**	0	–	−8.9
		**4b**	0	–	−10.8
		**4c**	1	Trp 30	−9.9
		**4d**	0	–	−10.4
		**4e**	1	Lys 253	−9.8
		**Nystatin**	2	Asn 338, Gln 341	−9.7
Transferase enzyme particularly SNF-1 protein kinase complex from *Saccharomyces cerevisiae*	3te5	**4a**	5	Ser 145, Gln 168, Ser 222, Ser 223, Arg 295	−8.6
		**4b**	3	Thr 81, Thr 82, Thr 83	−8.7
		**4c**	2	Thr 310, Ser 312	−9.1
		**4d**	4	Ser 145, Gln 168, Ser 222, Ser 223	−9.3
		**4e**	1	Arg 292	−9.3
		**Nystatin**	4	Thr 83, Asp 84, Lys 111, Arg 220	−9

Discussing the anti-bacterial results of the newly synthesized compounds' binding affinities to several bacterial proteins, it became clear that compounds 4b, 4c, and 4e outperformed tetracycline in the docking studies. Having higher binding affinity depicted the stability of the complex. The substance that had the strongest anti-bacterial action, however, was 4d. This substance not only remained stable in its binding pocket but also showed astounding pharmacological efficacy.

While in case of anti-fungal properties, nearly all of the compounds demonstrated high anticipated binding affinities for various fungal strains' proteins. With a predicted binding affinity of −10.3 kcal/mol, compound 4e was the best-scoring molecule against the Dihydrofolate Reductase enzyme isolated from Candida glabrata. This substance formed hydrogen bonds with Thr-58 and Ser-61 present in the binding pocket of dihydrofolate reductase enzyme, which explained its high affinity for this protein and offered a potential mechanism of action. With a binding affinity of −10.8 kcal/mol, higher than nystatin's binding affinity, compound 4b was the compound with the best score against Candida albican membrane protein. Compound 4d and 4e performed best against the binding pocket of transferase enzyme particularly SNF-1 protein kinase complex from *Saccharomyces cerevisiae* suggesting that these newly synthesized derivatives might play an important role in dysregulating fungal gene expression. However, the *in-vitro* activity of these compounds on fungal strains proved otherwise.

**Fig. 1:**
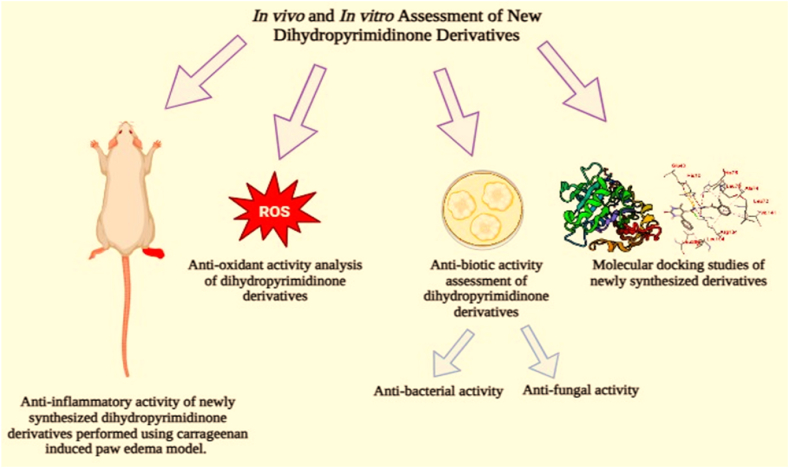
Graphical abstract representing the in-vitro and in-vivo experiments along with docking studies of the synthesized dihydropyrimidinones derivatives.

**Fig. 2:**
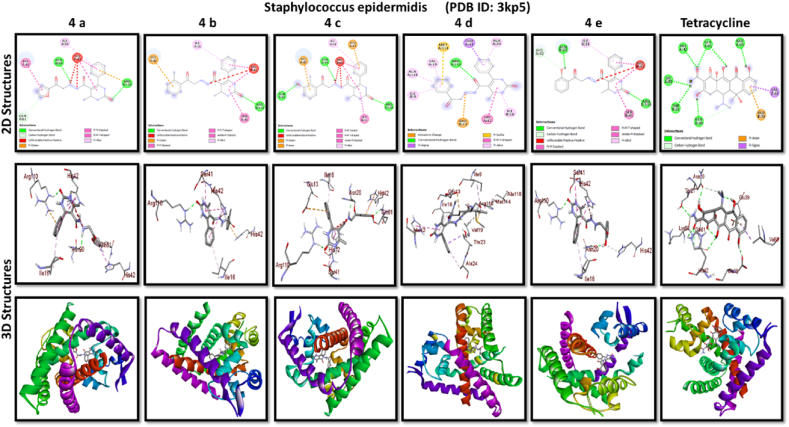

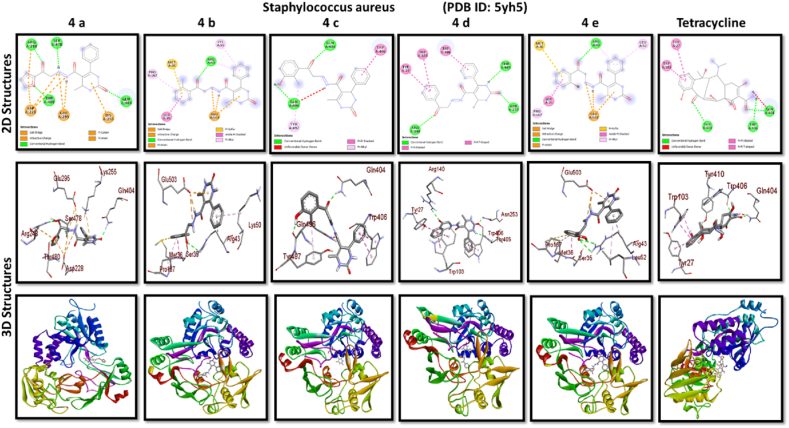

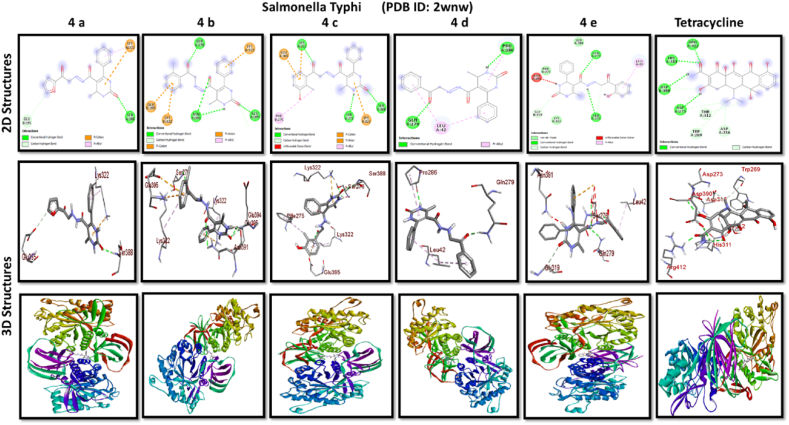

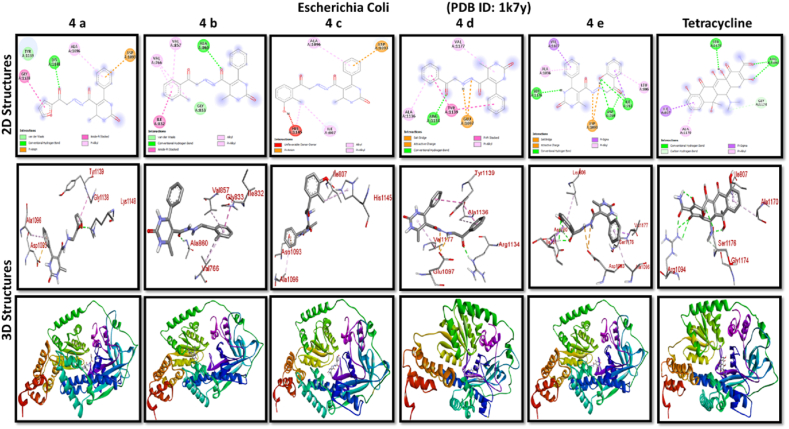

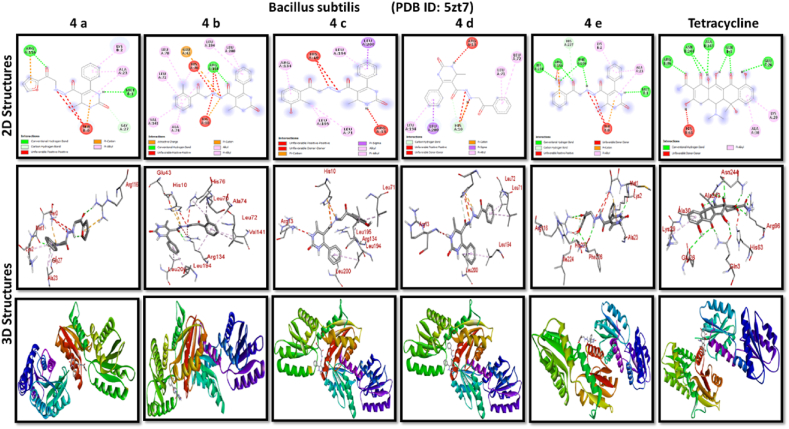

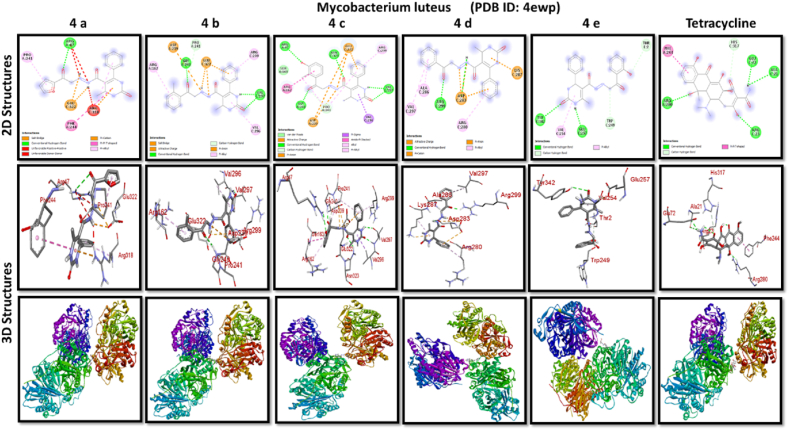

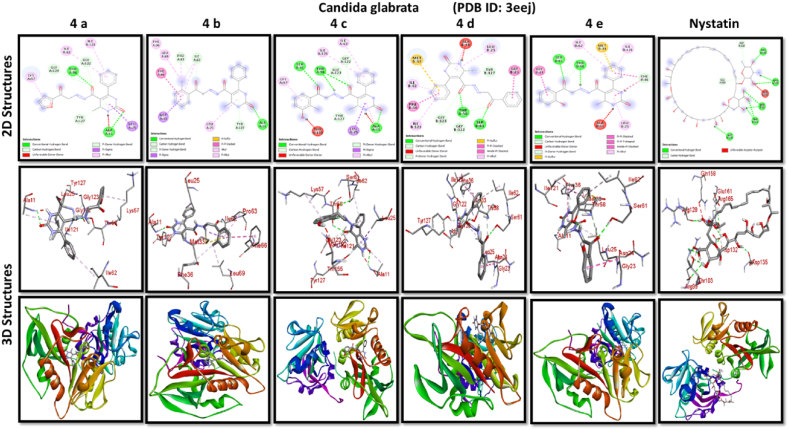

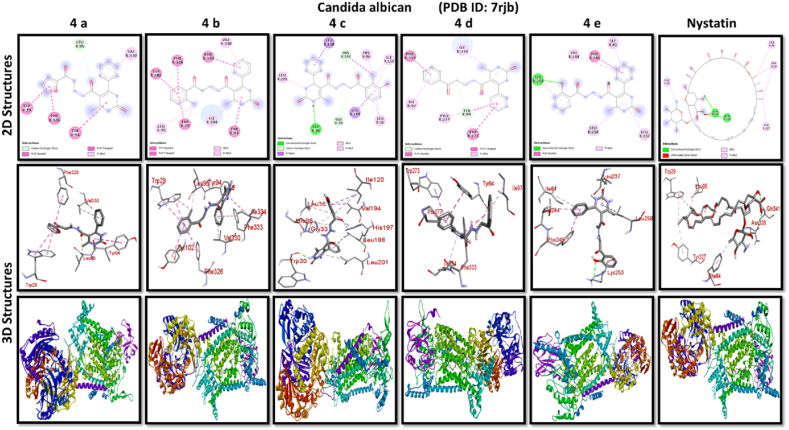

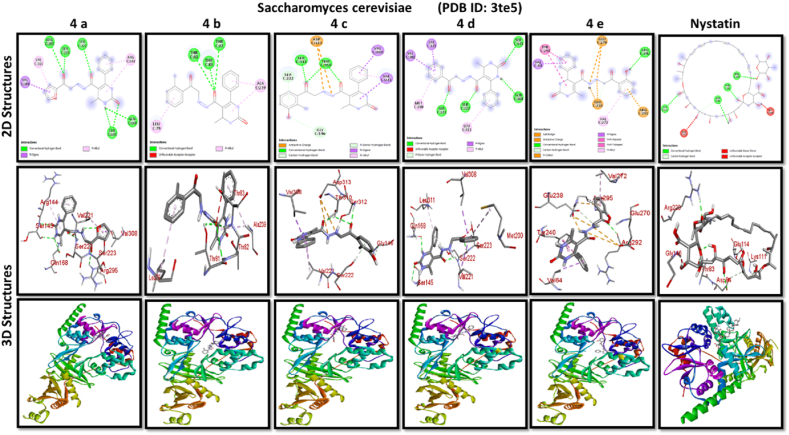
A: A post-dock analysis using the Biovia Discovery Studio Visualizer shows interactions between the compound 4a-4e against binding pocket of Staphylococcus epidermidis hindering transcription. B: A post-dock analysis using the Biovia Discovery Studio Visualizer shows interactions between the compound 4a-4e against binding pocket of oxidoreductase enzyme from *Staphylococcus aureus*. C: A post-dock analysis using the Biovia Discovery Studio Visualizer shows interactions between the compound 4a-4e against binding pocket of hydrolase enzyme from Salmonella Typhi. D: A post-dock analysis using the Biovia Discovery Studio Visualizer shows interactions between the compound 4a-4e against binding pocket of MetH C-terminal fragment form *Escherichia coli* resulting in structural alteration. E: A post-dock analysis using the Biovia Discovery Studio Visualizer shows interactions between the compound 4a-4e against binding pocket of sirohydrochlorin ferrochelatase (SirB)- a biosynthetic protein-form Bacillus subtilis leading to imbalance in bacterial cellular proteins. F: A post-dock analysis using the Biovia Discovery Studio Visualizer shows interactions between the compound 4a-4e against binding pocket of transferase enzyme form Mycobacterium luteus impacting fatty acid biosynthesis, ultimately leading to cellular death. G: A post-dock analysis using the Biovia Discovery Studio Visualizer shows interactions between the compound 4a-4e against hydrophobic binding pocket of dihydrofolate reductase enzyme form Candida glabrata. H: A post-dock analysis using the Biovia Discovery Studio Visualizer shows interactions between the compound 4a-4e against the binding pocket of a membrane protein from Candida albican. I: A post-dock analysis using the Biovia Discovery Studio Visualizer shows interactions between the compound 4a-4e against the binding pocket of transferase enzyme particularly SNF-1 protein kinase complex from *Saccharomyces cerevisiae* dysregulating fungal gene expression.

**Fig. 3:**
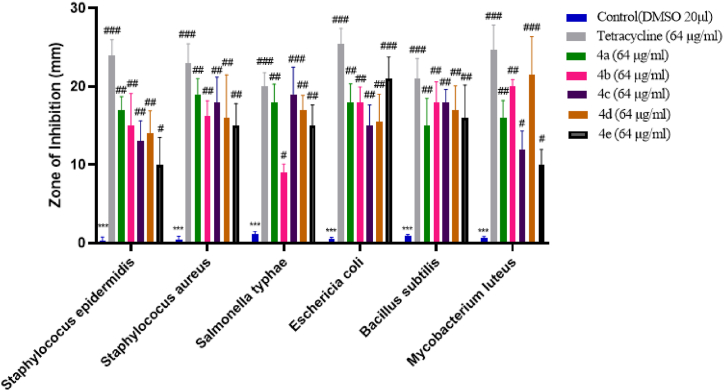
Anti -bacterial activity of synthesized compounds in terms of diameter of zone of inhibition (mm). Data was analyzed by mean ± SEM. ∗ Denotes a significant difference against the negative control; ^#, ##, ###^ show a significant difference against control. p < 0.05 is considered to be significant.

**Fig. 4:**
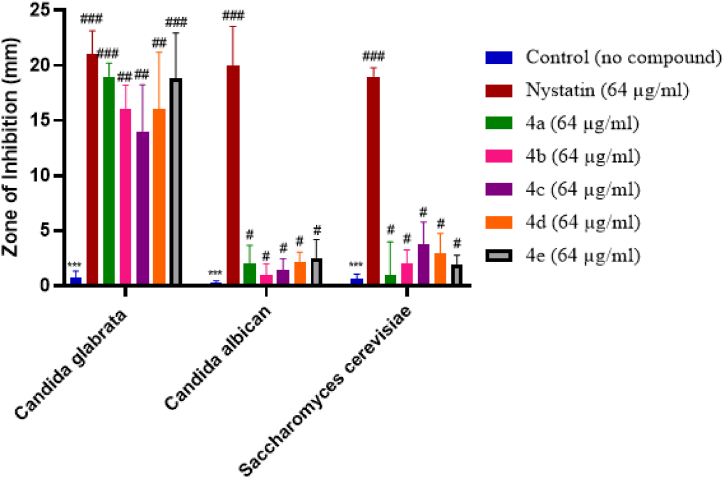
Anti-fungal activity of synthesized compounds in terms of diameter of zone of inhibition (mm). Data was analyzed by mean ± SEM. ∗ Denotes a significant difference against the control; ^#, ##, ###^ show a significant difference against control. p < 0.05 is considered to be significant.

All results of anti-bacterial and anti-fungal properties are presented in Table 1, Table 2 respectively (see Fig. 1).Fig. 1, Fig. 2, Fig. 3, Fig. 4

### 2.3 Anti-bacterial potential of dihydropyrimidinones derivatives (4a – 4e)

The antibacterial activity of all the dihydropyrimidinones derivatives **(**4a-4e**)** were assayed against six different bacterial strains: *Escherichia coli*, *Mycobacterium luteus, Staphylococcus aureus*, *Bacillus subtilis*, *Salmonella typhae,* and *Staphylococcus epidermidis*. All bacterial strains were cultured in agar at 37 °C overnight. The antibacterial properties of synthetic substances were tested in vitro on several bacterial strains and contrasted with negative control. Five replicates were taken in each case. Tetracycline was used as the gold standard medication. Diameter of zone of inhibition was then measured. Zone of inhibition was insignificant (∗∗∗p < 0.001) for negative control (dimethyl sulfoxide 20 μl). 4a was again found effective against staphylococcus epidermidis with a significance of (^##^p < 0.01 vs negative control), its effectivity was measured in terms of zone of inhibition which was 17 mm ± 1.74. Compound 4a performed well against *Staphylococcus aureus* (^##^p < 0.01 vs negative control). The diameter of zone of inhibition was measured to be 19 mm ± 2.00. For *Salmonella typhae*, dihydropyrimidinone derivative 4c showed significant zone of inhibition of 19.00 mm ± 3.50 compared to the negative control (^###^p < 0.001 vs negative control). Compound 4b was found to be least effective against *Salmonella typhae* (^#^p < 0.05 vs negative control). For *Escherichia coli*, compound 4e performed best with a significance of (^###^p < 0.001 vs negative control) with zone of inhibition as big as 21 mm ± 2.79 though it was still shorter than that formed by tetracycline. For *Bacillus subtilis* compound 4b and 4c performed best (^###^p < 0.001 vs negative control) with 17 mm ± 2.2. This was evident after observing zone of inhibition. Compound 4d were observed to have good zone of inhibition (21.56 mm ± 4.85) against *Mycobacterium luteus* with a significance of (^###^p < 0.001 vs negative control). Results are expressed in Fig. 2. At the end of this activity we can see that the compound 4b and 4c performed best with 17 mm ± 2.21 (see Fig. 3).

### 2.4 Anti-fungal potential of dihydropyrimidinones derivatives (4a – 4e)

According to the results of their in vitro antifungal activity, the newly synthesized compounds (4a – 4e) were found to be active against *Candida glabrata,* amongst the five newly formed compounds, 4a (19 mm ± 1.22) and 4e (18.81 mm ± 4.15) performed remarkably well with a significance of (^###^p < 0.001 vs negative control). Unlike nystatin which performed well against all three fungal strains, newly synthesized dihydropyrimidinone compounds failed to have any profound effect on *Candida albican* and *Saccharomyces cerevisiae* with a significance of (^#^p < 0.05 vs negative control). Finally, we can observe that for the anti-fungal activity against *Candida glabrata,* amongst the five newly formed compounds 4a showed best activity with 19 mm ± 1.22.

### 2.5 Anti-oxidant (DPPH assay) potential of dihydropyrimidinones derivatives

**Fig. 5:**
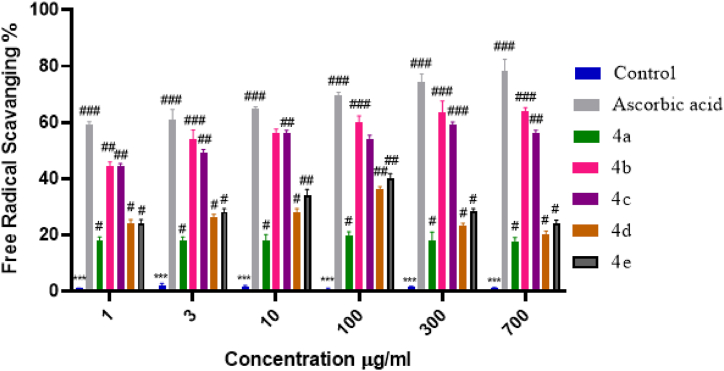
Anti-oxidant potential of synthesized compounds in terms of free radical scavenging effect (%). Data was analyzed by mean ± SEM. ∗ Denotes a significant difference against the control; ^#, ##, ###^ show a significant difference against control. p < 0.05 is considered to be significant.

The free radical DPPH is frequently used to assess a compound's capacity as a hydrogen source and free-radical scavenger. It is a well-known antioxidant and radical test in particular. The DPPH test depends on DPPH, a stabilized free radical, being eliminated. The DPPH radical initially displays a dark purple hue in solution; however, it becomes colorless or light yellow after reduction and transformation into DPPH-H. By using a DPPH assay, the synthesized dihydropyrimidinone derivatives (IVa-IVe) were assessed for their antioxidant capacity to scavenge free radicals. In comparison to the control DPPH solution, the sample solution's color intensity (measured in terms of absorbance) was assessed at 517 nm. All of the compounds, especially 4b, and 4c, showed potential radical scavenging action, as shown in the graph given below. It can be observed from the graph that the least-performing compound was 4a. It showed insignificant antioxidant activity of 19.81 ± 1.24 at a concentration of 100 μg/ml with a significance of ^#^p < 0.05 vs negative control. Compound 4b showed the highest radical scavenging activity in dose dependent manner. The antioxidant activity peaked (63.85 ± 1.39) at the concentration of 700 μg/ml, with a significance of (^###^p < 0.001 vs negative control). The second-best performing compound was 4c. At a concentration of 300 μg/ml, the scavenging activity of 4c was 59.11 ± 1.01 with a significance of (^###^p < 0.001 vs negative control). The activity of compound 4c gradually decreased with increased concentration. The overall antioxidant activity shown by the newly synthesized dihydropyrimidinone derivatives (4a-4e) was commendable. Ultimately it is concluded that the analogue 4b exhibited best anti-oxidant potential with 63.85 ± 1.39 (see Fig. 5).

### 2.6 *In-vitro* cytotoxicity results of the selected derivatives 4a & 4b by MTT assay

**Fig. 6:**
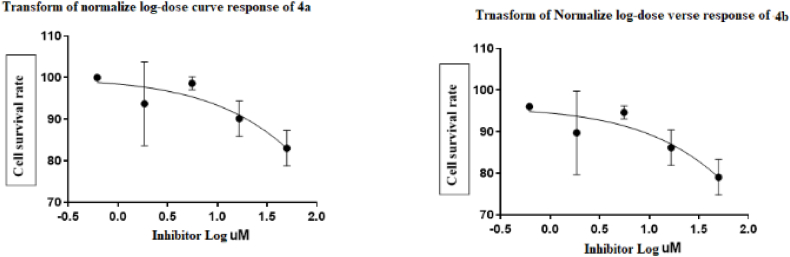
Cytotoxic effect of the selected derivatives 4a & 4b showing no toxicity against human cell lines and the cell survival rate is 99 % and 98 % respectively at the given dose of 2 μM.

The selected analogues 4a and 4b were screened for in-vitro cytotoxicity assay on human cell lines to see their toxic effects. The results showed non-toxic results and did not give 50 % inhibition of cells at the given concentrations. Even the cell survival rate was appreciated at particular concentrations. Thus it is concluded that these derivatives can be further used for *in-vivo* analysis on molecular level. The results are presented in Fig. 6.

### 2.7 Anti-inflammatory potential of dihydropyrimidinones derivatives

The literature has reported the biological significance of heterocyclic dihydropyrimidinones derivatives as anti-inflammatory agents. A study on the new 3,4-dihydropyrimidinone urea derivatives' anti-inflammatory effectiveness against IL-6 is one example. Tumor necrosis factor alpha (TNF-a) is inhibited by several dihydropyrimidinones. Despite their ease of synthesis, there has, to our knowledge, been no publication on the synthesis and assessment of the biological activity of 6-methyl-2-oxo-4-phenyl-1,2,3,4-tetrahydropyrimidine-5-carbohydrazide derivatives. In the current study, the effect of dihydropyrimidone derivatives (4a-4e) was investigated on reducing inflammation by inhibiting TLR signaling, which in turn led to the downregulation of proinflammatory cytokines.

The anti-inflammatory activity of the synthesized compounds was evaluated and recorded based on hind paw edema using albino mice as an experimental model. The results were observed by increase the paw thickness before and after induction of carrageenan injection, i-e, at 0 h and then 1,2,3,4,5, and 6th hour after injection. Results revealed that the test compounds with the dose of 200 mg exhibited good anti-inflammatory potential as compared to the dose level of 100 mg/kg. All of the compounds, especially 4a, and 4b, showed potential anti-inflammatory activity against carrageenan induced inflammation, as shown in the graphs given below. It can be observed from the graph that the least-performing compound was 4d. It showed insignificant anti-inflammatory activity, which peaked at the 3rd hour, 3.487 mm ± 3.62 at a concentration of 200 mg/kg with a significance of ^#^p < 0.05 vs negative control. This showed that the toxicity increased at higher dose level for compound 4d. Unlike former the compound 4a showed the highest anti-inflammatory activity in a dependent manner. It reduced the inflammation to 1.011 mm ± 0.247 at the concentration of 200 mg/kg, with a significance of (^##^p < 0.01 vs negative control). The second-best performing compound was 4b. At a concentration of 200 mg/kg, the anti-inflammatory activity of 4b was 1.447 mm ± 0.212 with a significance of (^##^p < 0.01 vs negative control). Overall, the newly synthesized dihydropyrimidinones derivatives (4a-4e) demonstrated significant anti-inflammatory action. Amongst them the compound 4a and 4b showed highest anti-inflammatory potential with 1.011 mm ± 0.247 mg/kg and 1.447 ± 0.212 mg/kg in countering inflammation in decreasing the inflammation in hind paw of the mice.

**Fig. 7:**
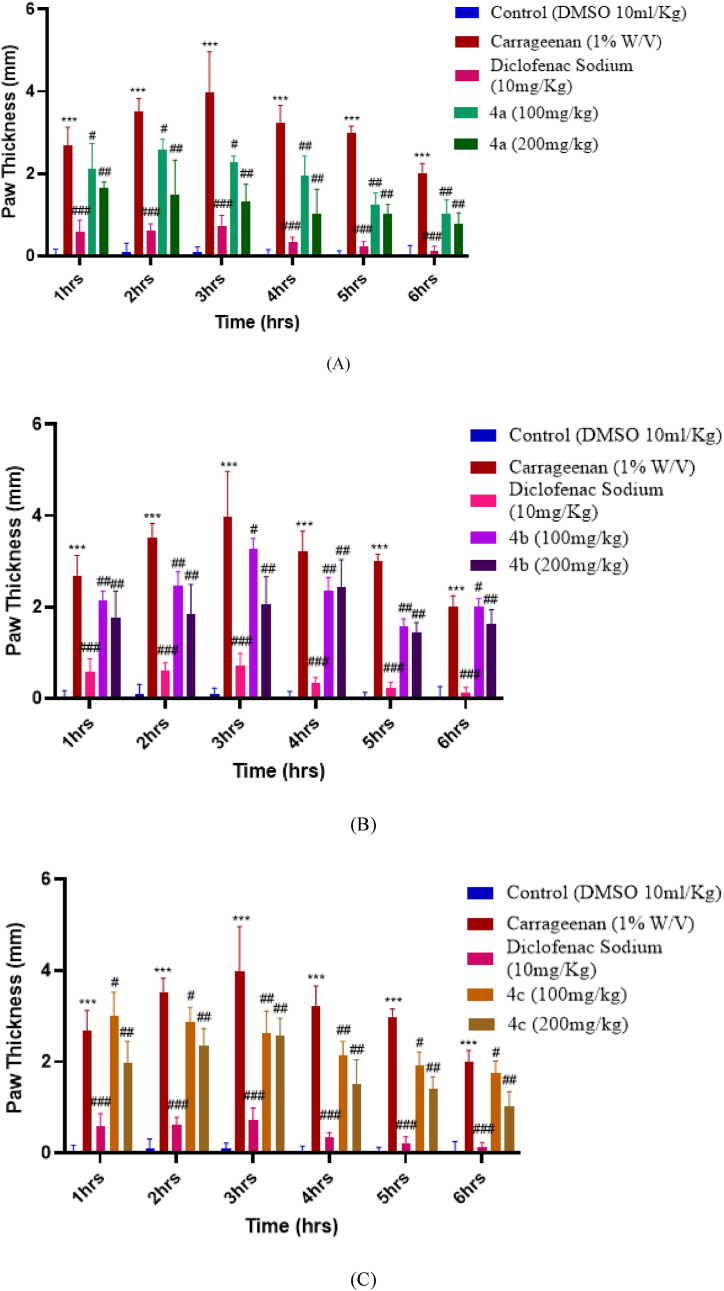

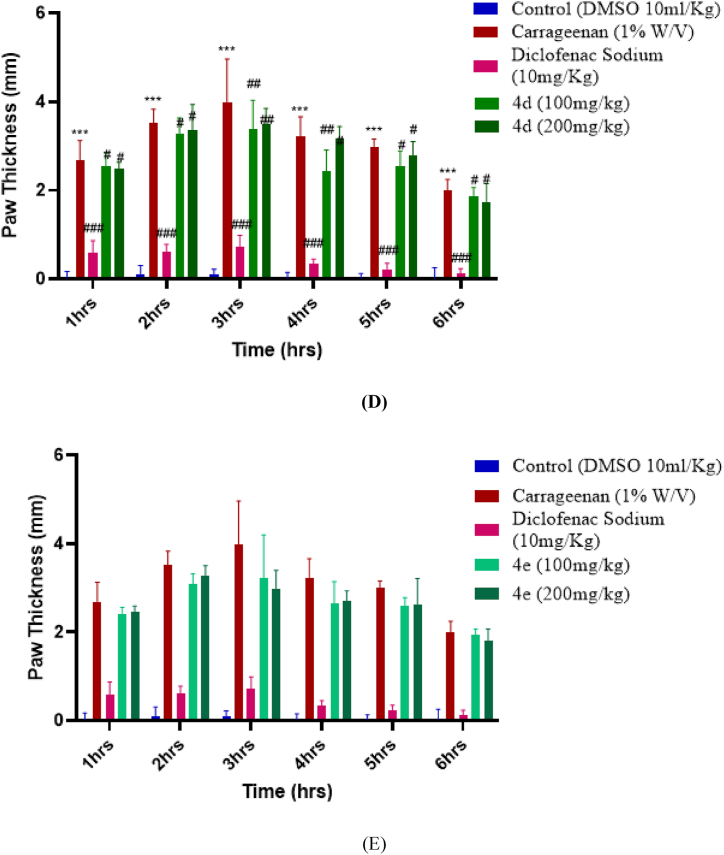
(A–E) Represents the anti-inflammatory potential of compounds (**a-e)** respectively.

Fig. 7: Anti-inflammatory activity of compound (A-E) respectively. Data was expressed in terms of ±SEM. P < 0.05 is considered as significant. ∗*p* represents the significant difference relative to the control group and ^#, ##, ###^*p* represents the significant difference relative to the disease group.

## 3 Conclusion

Inflammation is the major source of triggering multiple inflammatory mediators like COX-1 and COX-2 leading to ROS-associated oxidative stress. Bacterial and fungal infections are also linked with inflammation. From all the above experiments it was concluded that our newly synthesized dihydropyrimidinones derivatives revamp the inflammation associated with bacterial and fungal infection and lessen the oxidative effect. These derivatives also reduce the inflammation via toll like receptors (TLRs) along with non-toxic effects on human skin cell lines. We also claim that these derivatives could further use for different neuroinflammatory mediators and brain disorders like epilepsy and Alzheimer's disease. Finally, it is concluded that the recently synthesized new dihyropyrimidinone derivatives are highly effective antimicrobial derivatives with non-toxic effects on human skin cell lines.

## 4 Experimental section

### 4.1 General procedure for the synthesis of ethyl 6-methyl-2-oxo-4- phenyl-1,2,3,4-tetra hydropyrimidine-5-carboxylate (1)

A mixture of benzaldehyde (0.01 mol), urea (0.01 mol) and ethyl aceto acetate (0.013 mol) in 100 ml methanol was refluxed for 12 h in the presence of few drops of concentrated HCl used as catalyst. After completion of reaction mixture could cool at room temperature. Crude solid obtained was then recrystallized with ethanol and collected [13].

#### 4.1.1 General procedure for the synthesis of hydrazides (2)

Ethyl 6-methyl-2-oxo-4-phenyl-1,2,3,4-etrahydropyrimidine (I) was dissolved in ethyl alcohol (100 ml). Hydrazine hydrate (4mmoles) was added into the solution and reaction mixture was refluxed for 12hrs. (Completion of reaction was monitored by TLC) (1:2 ethyl acetate: pet ether). The white colored product obtained was filtered and recrystallized from ethanol [39].

#### 4.1.2 General procedure for the synthesis of hydrazones (4a -4e)

The carbohydrazide (1mmole) was dissolved in ethanol (30 ml) to get clear solution. Different substituted aryl aldehydes (1mmole) dissolved in ethanol were added to this solution and add acetonitrile: dioxane (3:1 v/v). The reaction mixture was refluxed for 36hrs. Completion of reaction was monitored by TLC) (1:2 ethyl acetate: pet ether). Crude Solid was obtained by evaporating the ethanol which was recrystallized using ethanol and dried [38].

Melting points of synthesized compounds was recorded by digital Gallan hamp (SANYO) model MDP. ^1^HNMR were analyzed by Bruker AV400 spectrophotometer in CD_3_ OD and CD Cl_3_ at 300 MHz. TMS was used as internal reference. The progress of reactions was monitored by the help of TLC. FT-IR spectra were recorded on alpha Bruker FT-IR spectrophotometer (Vmax in cm^−1^).

#### 4.1.3 Ethyl -6-methyl-2-oxo-4-phenyl-1,2,3,4-tetrahydropyrimidine-5-carboxylate (I)

Yield 95 %, m.p. 195–200. R_f_0.88. IR (KBR) cm^1^; 3326 (NH), 1700 (C=O ester), 1691, (C=O amide). ^1^HNMR (DMSO ppm), 1.12 (t, J = 7.5Hz, 3H), 2.2–2.30 (s, 3H), 4.00 (q, 7.5Hz, 2H), 5.11 (d, J = 3.0Hz, 1H), 7.25 (m, 4H, Ar-H), 9.19, (s, 1H, NH).

#### 4.1.4 6-methyl-2-oxo-4-phenyl-1,2,3,4-tetrahydropyrimidine-5-carbohydrazide (2)

Yield 98 %, m.p.180-185. White crystalline solid, R_f_ 0.72, (ethyl acetate: pet ether 2:1); IR (V_max_cm^−1^): 3232 (N-H), 1722 (C=C), 1697 (C=O amide), 1643 (C=O amide).

#### 4.1.5 5-(1-((2E)-2-furan-2-ylmethylidene) hydrazinyl)ethenyl)-6-methyl-4-phenyl-3,4-dihydropyrimidine-2(1H)-one (4 a)

Yield 40 %.m.p, 200–210 °C. R_f_ 0.55. IR (V_max_cm^−1^) 3228 (N-H), 2926 (C-H), 1720 (C=C), 1697 (C=O amide), 1643 (C=N), ^1^HNMR (DMSO ppm); 2.35 (s, 3H, CH_3_), 3.32 (s, 1H, NH), 3.61 (s, 1H, NH), 5.32 (s, 1H, CH), 7.25–7.33 (m, 5H, Ar-H).

#### 4.1.6 6-methyl-4-phenyl-5-(1-((2E)-2-(1-phenylethylidene)hydrazinyl)ethenyl)-3,4-dihydropyrimidine-2(1H)-one (4 b)

Yield 45 %.m.p, 194 °C. R_f_ 0.59. IR (V_max_cm^−1^) (NH), 2976 (C-H), 1720 (C=C), 1697 (C=O two amide peaks), 1645 (C=N), ^1^HNMR (DMSO ppm); 2.25 (s, 3H, CH_3_), 2.50–2.54 (s, 3H, CH_3_), 3.35 (s, 1H, NH), 3.53 (s, 1H, NH), 5.14–5.15 (s, 1H, CH), 7.23–7.75 (m, 5H, Ar-H), 9.20.9.23 (s, 1H, NH/OH).

#### 4.1.7 5-(1-((2E)-2-(2-hydroxybenzylidene)hydrazinyl)ethenyl)-6-methyl-4-phenyl-3,4 dihydro pyrimidine-2(1H)-one (4 c)

Yield 55 %, m.p. 195 °C.R_f_0.6.IR (KBR) cm^−1^. 3224 (NH), 2929 (C-H), 1720 (C=C), 1697 (C=O two amide peaks), 1643 (C=N), ^1^HNMR (DMSO ppm); 2.35 (s, 3H, CH_3_),3.32 (s, 1H, NH), 3.61 (s, 1H, NH), 4.05–4.07 (s, 3H, OCH_3_), 5.32 (s, 1H, CH), 7.25–7.33 (m, 5H, Ar-H), 9.76 (s, 1H, OH).

#### 4.1.8 5-(1-((2E)-2-(3-hydroxy-2-methoxybenzylidene)hydrazinyl)ethenyl)-6-methyl-4-phenyl-3,4- dihydropyrimidine-2(1H)-one (4 d)

Yield 40 %, m.p. 190–192, R_f_ 0.56. IR (KBR) cm^−1^.3232 (NH), 2927 (C-H), 1722 (C=C), 1697 (C=O two amide peaks), 1645 (C=N), ^1^HNMR (DMSO ppm); 2.35 (s, 3H, CH_3_), 2.50 (s, 1H, CH_3_), 3.34 (s, 1H, NH), 3.51 (s, 1H, NH), 5.14–5.15 (s, 1H, CH), 7.2–7.7 (m, 5H, Ar-H). 9.2–9.23 (s, 1H, NH).

#### 4.1.9 6-methyl-4-phenyl-5-(1-((2E)-2-(1-phenylethylidene) hydrazinyl)ethenyl)-3,4-dihydropyrimidine −2(1H)-one (4 e)

Yield 30 %, m.p. 188–189 °C, R_f_ 0.61. IR (KBR) cm^−1^. 3234 (NH), 2976 (CH_3_), 1720 (C=C), 1697 (C=O two amide peaks), 1645 (C=N), ^1^HNMR (DMSO ppm); 2.25 (s, 3H, CH_3_), 2.50–2.54 (s, 3H, CH_3_), 3.35 (s, 1H, NH), 3.53 (s, 1H, NH), 5.14–5.15 (s, 1H, CH), 7.23–7.75 (m, 5H, Ar-H), 9.20.9.23 (s, 1H, NH/OH).

#### 4.1.10 Molecular docking studies

To ascertain the potential binding affinities of newly synthesized dihyroprimidinone derivatives (4a - 4e) to target proteins of various bacterial and fungi strains, in-silico studies were conducted using Autodock Vina version 4.2.6. The X-ray crystal structure of the bacterial proteins used were the following: Transcription regulator from Staphylococcus epidermidis (PDB ID: 3kp5), oxidoreductase enzyme from *Staphylococcus aureus* (PDB ID: 5yh5), hydrolase enzyme from Salmonella Typhi (PDB ID: 2wnw), MetH C-terminal fragment form *Escherichia coli* (PDB ID: 1k7y), sirohydrochlorin ferrochelatase (SirB)- a biosynthetic protein-form Bacillus subtilis (PDB ID: 5zt7), and transferase enzyme form Mycobacterium luteus (PDB ID: 4ewp). Fungal proteins used for docking were following: dihydrofolate reductase enzyme form Candida glabrata (PDB ID: 3eej), membrane protein from Candida albican (PDB ID: 7rjb), transferase enzyme particularly SNF-1 protein kinase complex from *Saccharomyces cerevisiae* (PDB ID: 3te5). These structures were all retrieved from https://www.rcsb.org/pdb, an online protein data bank. To locate active binding pockets of targets, the DoG Site Scorer (ps://bio.tools/dogsitescorer) was utilized. Complexes of the ligand and protein were created for docking investigations. Before being stored in PDB format, the targeted proteins were cleaned by Biovia Discovery Studio Visualizer (DSV) to get rid of water and cocrystallized ligands [39]. Mol files were produced by drawing the structures of synthesized chemicals in Chemsketch. All ligand files were translated to PDB format using Open Babel. Additionally, PDBQT files of targets and ligands were produced using Autodock Tools version 1.5.6. Additionally, docking was done using the digital molecular docking software PyRx. Using Biovia DSV, the ideal structural poses and ligand-target molecular interactions were deciphered. To verify the docking method, the best poses of co-crystallized ligands and the best poses of re-docked configurations were compared.

### 4.2 *In-vitro* assay

#### 4.2.1 Anti-bacterial activity of the synthesized compounds (4a-4e)

Bactericidal activity was carried out by using ager well diffusion method [39] with small changes. Standard inoculum of the test microorganisms was utilized to inoculate agar plates. The test substances were then put in the desired concentration on 6 mm-diameter filter paper discs, which were then placed on the agar surface and gently pressed down to ensure complete contact of the disc with the agar surface. The petri dishes were incubated at 37 °C for 24 h. The test microorganism's germination and growth were typically inhibited by the antimicrobial agent, which in this particular case were the dihydropyrimidinone derivatives (4a-4e) that diffused into the agar. The diameters of the inhibitory growth zones were then determined. The inhibition zones obtained were compared with positive control. Tetracycline served as standard drug. For the negative control, discs were saturated with 20 μl of dimethyl sulfoxide. Different bacterial strains, *Staphylococcus epidermidis*, *Staphylococcus aureus* (Gram positive), *Mycobacterium luteus*, *Salmonella typhi, Bacillus subtilis, Escherichia coli* (gram negative), were used for the given test. Five replicates were taken in each case.

#### 4.2.2 Anti-fungal activity of the synthesized compounds (4a-4e)

Fungi derived from marine showing promising sources of structurally resembled and novel natural bioactive molecules. The focus of this finding was to develop anti-inflammatory drugs [40]. New cyclopeptides from antagonistic metabolites were also generated by marine-derived fungus [41]. In this study *Candida glabrata, Candida albican*, and *Saccharomyces cerevisiae* growth were investigated for the antifungal efficacy of compounds 4a - 4e. By adjusting the substances in potato dextrose agar (PDA) medium, the impact of newly synthesized dihyropyrimidinone derivatives (4a - 4e) and nystatin (acting as positive control) on the radial growth of three fungi species was evaluated. DMSO was used to create stock solutions of the substances under investigation. To achieve the final concentration of 64 μg/ml, each drug and nystatin from the stock solution were separately added to the melted, sterilized PDA medium in conical flasks. Each 20 mL PDA medium containing the compounds at the aforementioned concentration was properly stirred before being put into a 90 mm Petri plate. Petri dishes containing no compound were used as a control. Three duplicates of each sample were obtained. The diameter of the inhibitory zone was measured and compared with the control after the petri plates had solidified. Each petri plate was then individually inoculated aseptically with mycelial discs from pure cultures for 48 h at 25 ± 1 °C. zone of inhibition were then measured and compared with control.

#### 4.2.3 2, 2-diphenyl-1-picrylhydrazyl assay (DPPH assay)

Anti-oxidant activity of synthesized compounds was performed by using 2, 2-diphenyl-1-picrylhydrazyl (DPPH) as described by Ref. [42]. The stock solution of all Samples having a concentration of 1mg/10 ml was prepared. DPPH solution (0.004 % w/v) was prepared. Ascorbic acid (vitamin C) was used as standard antioxidant and DPPH was used as blank. Different concentrations (1,3, 10 100 300, and 700 μg/mL) of test compounds were pipetted to the test tubes and the volume was adjusted to 3 mL with methanol. 1 mL of DPPH (0.1 mM) solution was mixed with 1 mL of sample and standard solution separately. The antioxidant activity is inversely proportional to the absorbance. The working solutions of test compounds and standards were prepared in methanol. The samples were vortexed, and incubated in the dark at room temperature for 30 min and the absorbance was measured at 517 nm against blank samples in a spectrophotometer (The absorbance was recorded, and radical scavenging activity was expressed as percentage inhibition of DPPH radical and was calculated by the following equation:

$$Scavenging\;effect\;\left(\%\right)=\frac{\left(Absorbance\;of\;negative\;control-Absorbance\;of\;test\;sample\right)}{Absobance\;of\;negative\;control\;}\times 100$$

#### 4.2.4 Cell lines and cell culture

A human non-cancer keratinocytes cell lines HaCaT were gained from Operational Laboratories Kahuta Research Laboratories, Islamabad, Pakistan. Research resource ident Research Resource identifier (RRID) as in ExPASY Cellulose for the human non-cancer keratinocytes cell lines was found to be RRID: CVCL_0038). These cell were maintained and cultured and supplemented with 10 % FBS, 2m M L-glutamine, 1m M Na-pyruvate, 100U/m L penicillin, 100 μg/m L streptomycin at 37 °C in a humidified 5 % CO _2_ atm [43].

#### 4.2.5 Cell line authentication method (STR analysis)

STR loci was selected for cell line authentication because they showed the maximum possibility of variation in discrimination amongst human cell lines with the fewest PCR targets and they can easily detect cross contaminating cells. STR profile of cell lines was compared with those tissue samples at the genome level to check whether they are derived from original donor. To perform STR genotyping, PCR primers were designed to enlarge every selected STR loci so that every allele can be distinguished by their sizes. One of the primer of each pair was labelled with fluorescent dye. The size range for each STR locus was determined by number of variants that differ in length [44]. The authentication of cell lines was carried out in the month of September −2020.

#### 4.2.6 Detection of mycoplasma in cell culture

To test a culture, make sure that the growth medium is free from antibiotics. Remove all about 3 ml of fluid from dense culture which has not been fed for at least three days. Scrapped off monolayer and saturated suspension culture may be sampled directly. Incubate 1 ml of cell suspension in a tube of mycoplasma broth and 0.1 ml into the plate of mycoplasma ager. Incubate the broth and ager at 37 °C in humidified 95 % nitrogen, 5 % CO _2_. A change in the pH of the broth or turbidity warns the mycoplasma contamination. Following 14days after one week transfer 0.1 ml of broth in fresh plate of mycoplasma ager and incubate at 37 °C anaerobically. Using inverted microscope all the ager plates were examined at 100 X and 300 X for the next three weeks [45].

#### 4.2.7 In*-vitro* cytotoxicity study by MTT assay on human HaCaT cell lines

The cytotoxic effect of the selected compounds 4a & 4b in human non-cancer keratinocytes cell lines HaCaT was determined by MTT (3-(4, 5-dimethylthiazol-2-yl)-2,5-diphenyltetrazolium bromide [43]. Cells (1x10 ^5^/well) were placed in 0.2 ml of medium in 96-well plates and were kept for 36hr in CO2 incubator. For MTT assay, after incubation, the medium was removed carefully from the wells. MEM (w/o) and FCS was used to wash the each well 2–3 times and 200 μl of MTT (5 mg/ml) was added. 1 ml of DMSO was added to each well and mixed well with the help of micropipette and then left it for 45 s. Presence of viable cells were visualized by the development of purple colour due to the formation formazan crystals. Concentration required for 50 % inhibition of viability (IC_50_) was determined graphically. Graph was plotted taking concentration on X-axis and relative cell viability at Y-axis.

Cell viability (%) = Mean OD/ control OD x 100

### 4.3 *In-vivo* assay

#### 4.3.1 Animals

Adult albino mice 10-12-week age (male/female) were used during the experimental work were taken from the Pharmacology lab of Riphah Institute of Pharmaceutical Sciences, Riphah International University (RIU) Islamabad, Pakistan. All mice were acclimated to the typical laboratory conditions of humidity (55 %) and temperature (22 ± 1 °C), as well as a 12-h light/dark cycle, with access to food and water as required. Every procedure was done as per the strict protocols set by the research and ethical committee (REC) Riphah International University (Ref. No.: REC/RIPS/2021/02).

#### 4.3.2 Carrageenan induced paw edema model

The carrageenan induced-hind paw edema model was performed by using the following method [44] with slight modifications. Prior to usage, carrageenan was prepared as a 1 % W/V solution in 0.9 % saline. To avoid the hypodermic needle bore becoming blocked, a complete solution of solid substance was created. Based upon the cytotoxicity studies of the dihydropyrimidinones derivatives the multiple doses (100 mg/kg, & 200 mg/kg) were selected to carry on the further studies. There were 13 groups of mice in this experiment. Each group contained 5 mice. The groups were split into:

(I) Vehicle Control (n = 5) was injected 10 ml/kg of 0.4 % DMSO subcutaneously;
(II) Negative Control (n = 5) was administered with 0.1 ml Carrageenan prepared as a 1 % W/V solution via subcutaneous route;
(III) Positive control (n = 5) was administered Diclofenac sodium, 10 mg/kg, via subcutaneous route;
(IV) (IV – VIII) 5 Treatment groups (n = 5 each) based upon the number of synthesized compounds. Same doses of these newly synthesized dihydropyrimidinone analogues were administered (100 mg/kg, via subcutaneous route).
(V) (VIII - XIII) 5 Treatment groups (n = 5 each) based upon the number of synthesized compounds. (200 mg/kg, via subcutaneous route) dose of newly synthesized dihydropyrimidinone analogue was administered.

For identifying purposes, an indelible pen was used to mark the tail of each animal. Animals received test substances 30 min before receiving a carrageenan injection. Diclofenac sodium (10 mg/kg), used as a positive control, was compared to the effects of the newly synthesized dihydropyrimidinone derivatives (4a-4e). Just before injecting carrageenan, the volume of the pre-injection paw was measured. The plantar portion of the left hind paw received a subcutaneous injection of 0.1 ml of a 1 % solution of lambda carrageenan prepared in 0.9 % saline. Animals that had been intraperitoneally administered 20 mg/kg of ketamine hydrochloride were gently sedated before injections were given. Using a plethysmometer, the paw thickness (mm) of the Carrageenan, control - both positive and negative - test compound (4a-4e) injected groups was measured hourly from 1 to 6 h. Results were later plotted in the form of a graph.

### 4.4 Statistical analysis

The analysis was done using Graph Pad Prism 8.0 and consisted of a one-way analysis of variance (ANOVA) followed by a post hoc Tuckey's test. The data were expressed as mean ± SEM. A two-way grouping analysis was also conducted. When compared to the negative control group, the symbol # indicates relevance. The data were deemed statistically significant at a p value of 0.05.

## Limitations and strength

### Limitations

No in-vivo studies on based on molecular level.

### Strengths

The dihydropyrimidinones derivatives have a great effect on bacterial and fungal strains along with in-vivo anti-inflammatory results, also the compounds shown non-toxic effect on human cell lines.

## CRediT authorship contribution statement

**Madiha Kanwal:** Writing – original draft, Methodology, Data curation. **Humaira Nadeem:** Supervision. **Sumra Malik:** Formal analysis, Data curation. **Saima Maqsood:** Methodology.

## Data availability

The data will be available on demand.

## Ethical statement:

The research work described in this manuscript has not been published previously nor under consideration in any journal. This article has approved by all authors technically and ethically. If this manuscript gets accepted and published in this journal, it will not be submitted again in any other journal for publication.

During the experimental work all procedures were carried out as per the guidelines approved by Research and Ethical Committee (REC) Riphah International University (Approval ID: Ref: No. REC/RIPS/2021/02). All animals were kept under standard environmental conditions (25 ± 2 °C) in polypropylene cages under light and dark cycle for 12 h. Food was mad available at libitium.

## Funding

There is no external funding available for this research.

## Declaration of competing interest

The authors declare that they have no known competing financial interests or personal relationships that could have appeared to influence the work reported in this paper.
